# Physiological change alters endophytic bacterial community in clubroot of tumorous stem mustard infected by *Plasmodiophora brassicae*

**DOI:** 10.1186/s12866-020-01930-4

**Published:** 2020-08-06

**Authors:** Diandong Wang, Tingting Sun, Songyu Zhao, Limei Pan, Hongfang Liu, Xueliang Tian

**Affiliations:** 1 Yangtze Normal University, Fuling, Chongqing, China; 2 Henan engineering research center of biological pesticide & fertilizer development and synergistic application, Henan Institute of Science and Technology, Xinxiang, Henan China

**Keywords:** Endophytic bacterial community, *Plasmodiophora brassicae*, Tumorous stem mustard, High-throughput sequencing, Physiological change

## Abstract

**Background:**

Endophytic bacteria are considered as symbionts living within plants and are influenced by abiotic and biotic environments. Pathogen cause biotic stress, which may change physiology of plants and may affect the endophytic bacterial communiy. Here, we reveal how endophytic bacteria in tumorous stem mustard (*Brassica juncea* var. tumida) are affected by plant physiological changes caused by *Plasmodiophora brassicae* using 16S rRNA high-throughput sequencing.

**Results:**

The results showed that Proteobacteria was the dominant group in both healthy roots and clubroots, but their abundance differed. At the genus level, *Pseudomonas* was dominant in clubroots, whereas *Rhodanobacter* was the dominant in healthy roots. Hierarchical clustering, UniFrac-weighted principal component analysis (PCA), non-metric multidimensional scaling (NMDS) and analysis of similarities (ANOSIM) indicated significant differences between the endophytic bacterial communities in healthy roots and clubroots. The physiological properties including soluble sugar, soluble protein, methanol, peroxidase (POD) and superoxide dismutase (SOD) significantly differed between healthy roots and clubroots. The distance-based redundancy analysis (db-RDA) and two-factor correlation network showed that soluble sugar, soluble protein and methanol were strongly related to the endophytic bacterial community in clubroots, whereas POD and SOD correlated with the endophytic bacterial community in healthy roots.

**Conclusions:**

Our results illustrate that physiologcial changes caused by *P. brassicae* infection may alter the endophytic bacterial community in clubroots of tumorous stem mustard.

## Background

Endophytic bacteria are symbionts living within plants for the majority of their life cycle without any negative effects on a host plant [1, 2]. It is well known that endophytic bacteria are beneficial to plant growth and development because they synthesize plant hormones (indole-3-acetic acid), solubilize phosphate and promote plant tolerance to biotic and abiotic stresses [3–5] by producing siderophores, competing with pathogens for space and nutrients, and modulating the plant resistance response [6, 7]. Moreover, some endophytic bacteria provide biologically-fixed nitrogen to host plants [8, 9].

Endophytic bacteria often live in plant intercellular spaces, where they easily absorb carbohydrates, amino acids, and inorganic nutrients [8, 10, 11]. When endophytic bacteria survive in the intracellular environment, they must adapt to that environment and be compatible with a host. This specific niche within host plants results in endophytic bacteria having fewer competitors. However, pathogens in infected plants would compete with endophytic bacteria for space and nutrients. In diseased plants, pathogens become the dominant microorganisms and fight with endophytic bacteria as well as plant. For example, the endophytic bacterial community in grapevine and apple infected by phytoplasmas [12, 13] and in tomato infected by root knot nematode [14] changed compared with healthy plants. In particular, pathogens alter plant physiolocial process and may indirectly affect the endophytic bacteria. However, which physiological changes may modify endophytic bacteria and how is unclear.

Clubroot is a serious disease of cruciferous crops caused by biotrophic *P. brassicae* Woronin [15], significantly changing morphology and physiology of the diseased plant, finally forming galls (i.e. clubroots) [16]. *Plasmodiophora brassicae* survives and absorbs carbohydrates in galls [17, 18], thus they occupy most space in root cells and probably suppress endophytic bacteria. However, how clubroot disease influences endophytic bacterial communities in tumorous stem mustard is unclear. The objectives of our study were (1) to reveal the species abundance in the endophytic bacterial community in clubroot (α-diversity), and (2) to compare the endophytic bacterial communities in clubroots and healthy roots (β-diversity), (3) to uncover how *P. brassicae* shapes the endophytic bacterial community through physiological changes in clubroots compared to healthy roots of tumorous stem mustard.

## Results

### α-diversity analysis

**Fig. 1:**
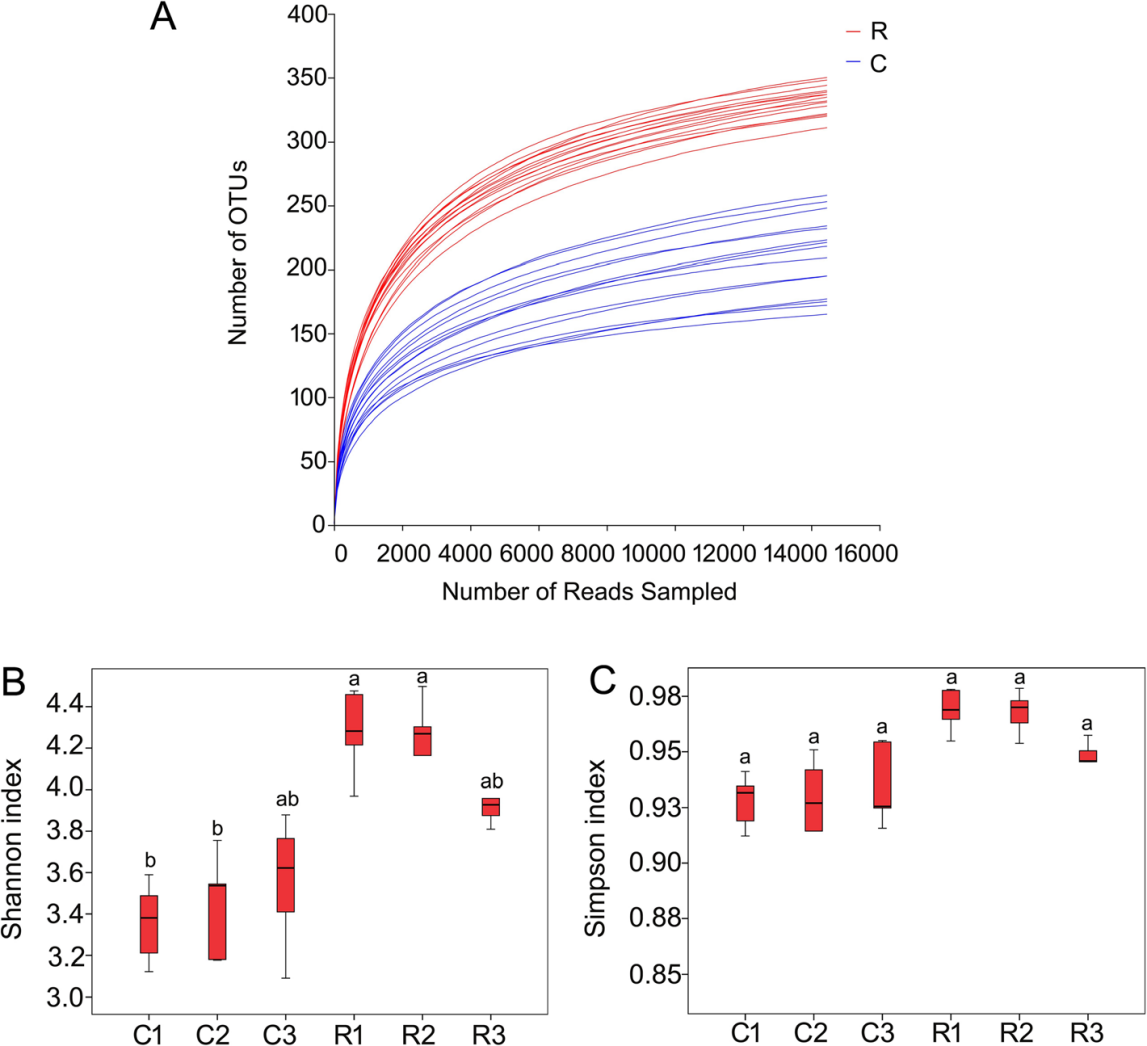
α-diversity of the endophytic bacterial communities in healthy roots and clubroots. **a** Rarefaction curves. **b** Shannon index. **c** Simpson index. R, healthy roots. C, clubroots. Different letters on the column showed the significant difference between healthy roots and clubroots

High quality sequences of partial 16S rRNA were produced by a Miseq PE3000 platform. The raw sequencing data have been deposited at the Sequence Read Archive (SRA, https://www.ncbi.nlm.nihgov/sra) under accession number PRJNA631176. According to the taxonomy of the sequences and abundance (Additional file 1:Table S1), we analyzed the composition of the endophytic bacterial community. Rarefaction curves analysis confirmed that the number of Operational taxonomic units (OTUs) increased asymptotically with an increase in reads (Fig. 1a). The rarefaction curves and Shannon index of the endophytic bacterial community in healthy roots were higher than those in clubroots, showing that healthy roots possessed more diverse community (Fig. 1a, b). However, the Simpson index showed no significant difference between healthy roots and clubroots (Fig. 1c).

**Fig. 2:**
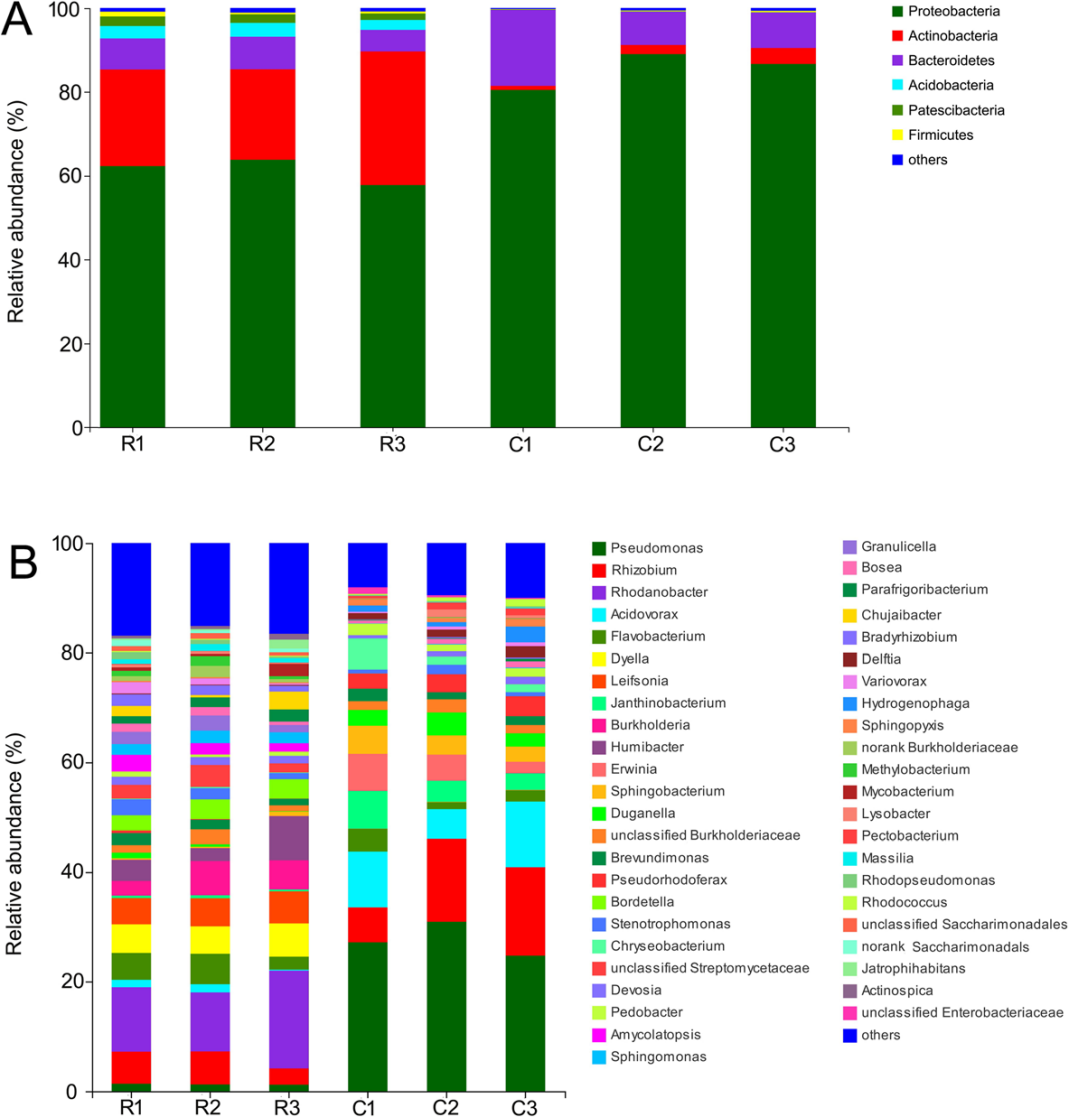
Distribution of endophytic bacteria at the phylum (**a**) and genus (**b**) level. R, healthy roots. C, clubroots

At the phylum level, Proteobacteria was the dominant group in healthy roots (relative abundance ranging from 57.8 to 63.8%) and in clubroots (relative abundance ranging from 80.4 to 89.0%) (Fig. 2a). Actinobacteria in healthy roots were the second abundant bacterial group with relative abundance ranging from 21.6 to 31.8%. However, the second abundant bacterial group in clubroots was Bacteroidetes (relative abundance ranging from 8.0 to 18.2%). At the genus level, *Rhodanobacter* (relative abundance ranging from 10.7 to 17.8%) was dominant in the endophytic bacterial community in healthy roots, followed by *Rhizobium*. However, *Pseudomonas* (relative abundance ranging from 24.7 to 30.9%) in clubroots was the dominant group, followed by *Rhizobium* and *Acidovorax* (Fig. 2b).

### β-diversity analysis

**Fig. 3:**
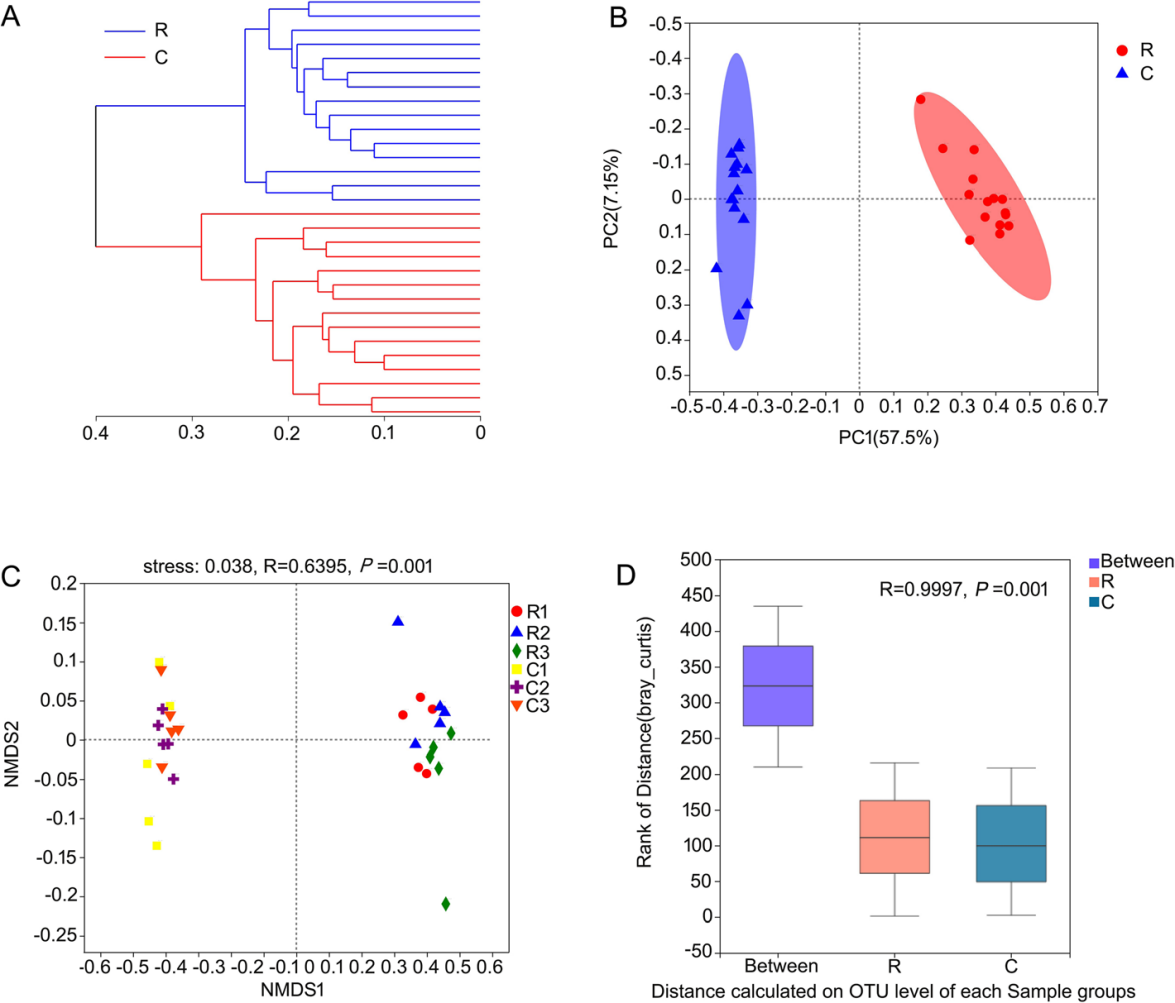
β-diversity analysis of the endophytic bacterial communities in the healthy roots and clubroots. **a** Hierarchical clustering analysis. **b** UniFrac-weighted PCA. **c** NMDS. **d** ANOSIM. R, healthy roots. C, clubroots

The endophytic bacterial community in the healthy roots and clubroots clustered in two branches on the hierarchical clustering tree (Fig. 3a). UniFrac-weighted PCA showed variations between the healthy roots and the clubroots with the first two axes explaining 57.5 and 7.2% of the total variation (Fig. 3b). The endophytic bacterial community in healthy roots was clustered on the right side of PCA, whereas the communities in the clubroots were clustered on the left side, indicating a clear separation between the communities in healthy roots and clubroots samples. Likewise, NMDS results with stress 0.038 also showed the same trends between the communities in healthy roots and clubroots (Fig. 3c), although some samples exhibited differences among three fields in one group such as healthy roots or clubroots. The results of ANOSIM with R 0.997 demonstrated the communities in healthy roots and clubroots significantly differed (Fig. 3d). The network analysis reflected that healty roots had a more complex endophytic bacterial community (Degree 3140 and Clustering 66.53) than clubroots (Degree 2632 and Clustering 58.77) (Additional file 2: Figure S1).

**Fig. 4:**
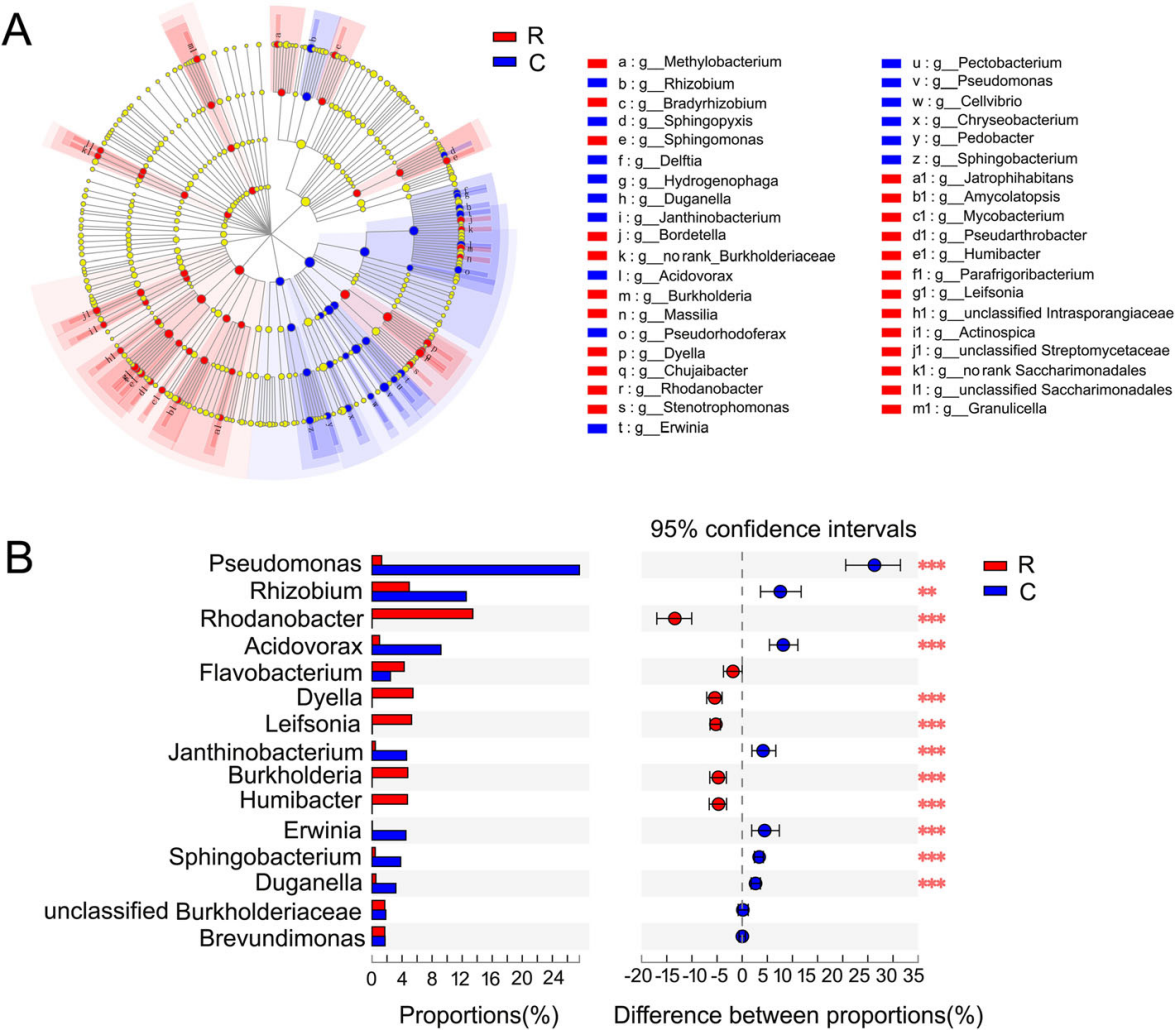
The markedly different bacteria in the endophytic bacterial communities between healthy roots and clubroots. **a** LefSe analysis. The cladogram shows the taxa with marked differences in the two endophytic bacterial communities. Red and blue indicate different groups, with the classification of taxa at the level of class, order, family, and genus shown from inside to the outside. The red and blue nodes in the phylogenetic tree represent taxa that play an important role in the two endophyte communities. Yellow nodes represent taxa with no significant difference. **b** Student’s *t*-test bar plot of the endophytic bacterial communities at the genus level in healthy roots and clubroots. *p* < 0.05*, *p* < 0.01**, *p* < 0.001***. R, healthy roots. C, clubroots

Significantly different taxa were found between the two communities based on the discriminant analysis effect size (LEfSe). At the genus level, *Methylobacterium*, *Bradyrhizobium*, *Sphingomonas*, and *Bordetella* were enriched in healthy roots and *Duganella*, *Rhizobium*, *Hydrogenophaga* and *Sphingopyxis* were biomarker species (Fig. 4a). Furthermore, the 15 most abundant genera of the two communities were compared by the Student’s t-test (Fig. 4b). *Pseudomonas* and *Rhizobium* were significantly more abundant in clubroots, whereas *Rhodanobacter* were markedly more abundant in healthy roots (Fig. 4b).

### Relationship between physiological properties and the endophytic bacteria community in healthy roots and clubroots

**Fig. 5:**
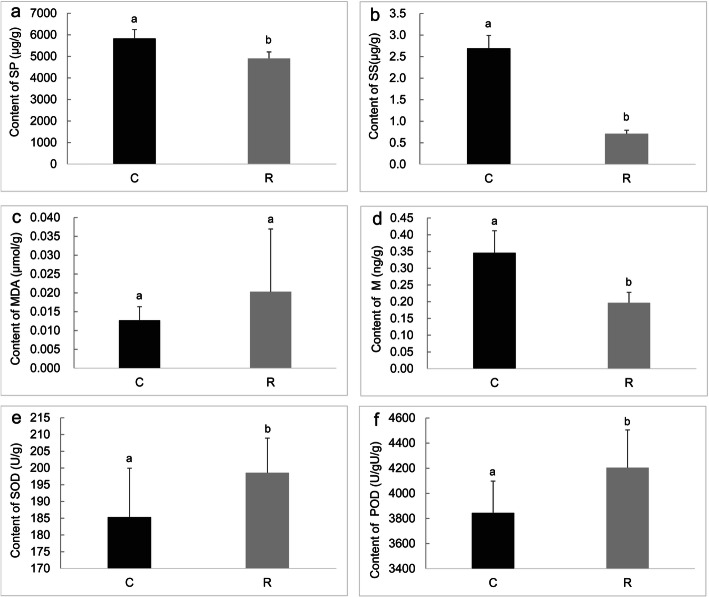
Comparision of physiological properties between healthy roots and clubroots. SS, soluble sugars. SP, soluble protein. M, methanol. MDA, malondialdehyde. POD, peroxidase. SOD, superoxide dismutase. Different letters on the column showed the significant difference between healthy roots and clubroots

**Fig. 6:**
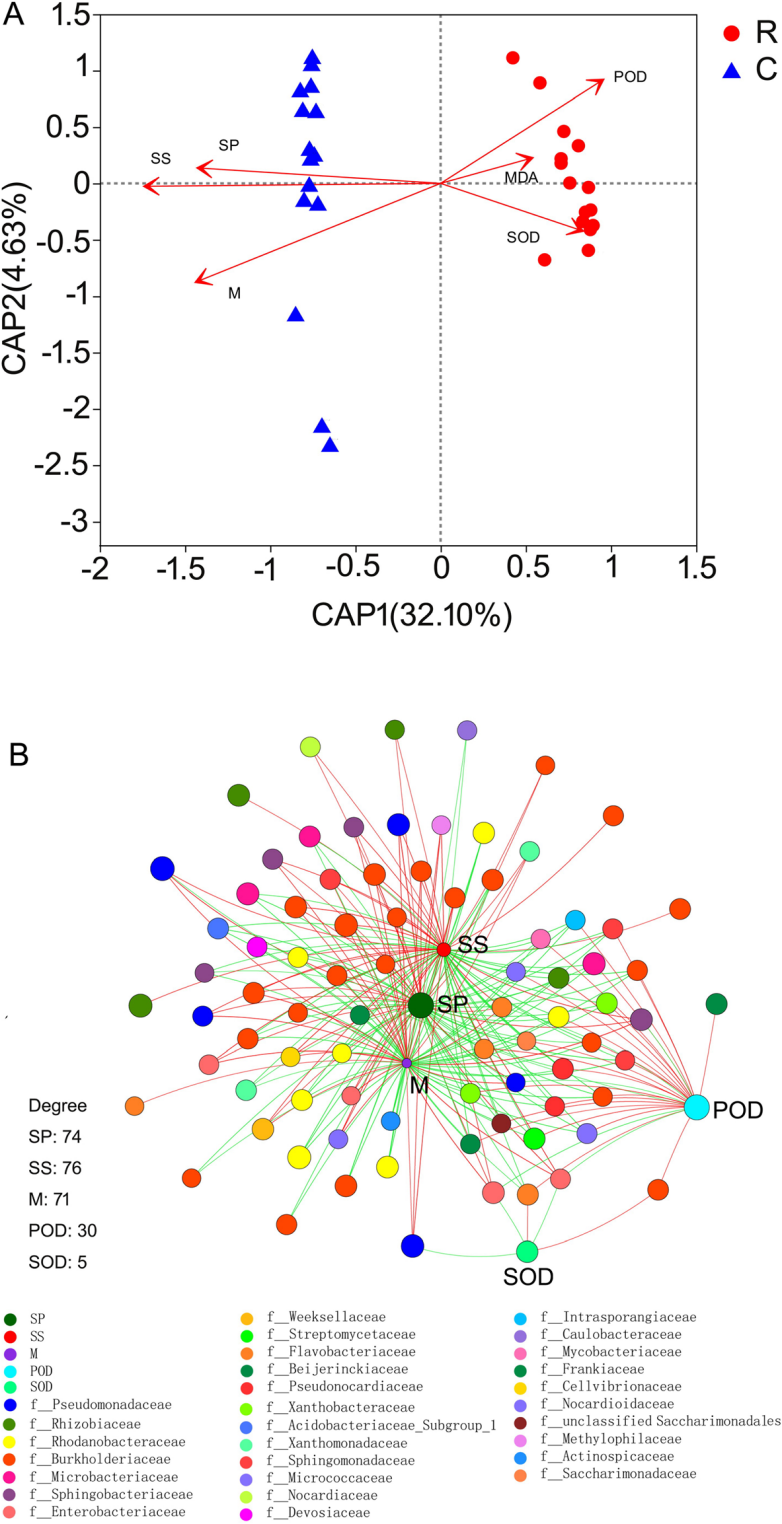
Relationship between physiological properties and the endophytic bacterial communities in healthy roots and clubroots. **a** db-RDA. SS, soluble sugars. SP, soluble protein. M, methanol. MDA, malondialdehyde. POD, peroxidase. SOD, superoxide dismutase. **b** Two-factor correlation network. The number represented the quantities of bacteria markedly correlated with physiological properties. A red line indicates a positive correlation, and a green line indicates a negative correlation

The physiological properties, such as soluble sugar, soluble protein, POD, SOD, and methanol in healthy roots and clubroots were markedly different, except for malondialdehyde (Fig. 5). Futhermore, we analyzed the relationship between physiological properties and the endophytic bacterial community. The results of d-b RDA showed that soluble sugar, soluble protein and methanol were strongly related to the community in clubroots, whereas POD and SOD correlated with the community in healthy roots (Fig. 6a). Moreover, we constructed two-factor correlation network and found that physiological properties correlated with some endophytic bacteria (Fig. 6b). For example, soluble sugar, soluble protein and methanol were related to endophytic bacteria with values of 76, 74 and 71, respectively, suggesting that they play important role in shaping the endophytic bacterial community in clubroots.

## Discussion

In our study, we found that the endophytic bacterial communities in healthy roots and clubroots differed markedly in alpha diversity and beta diversity. The dominant bacteria in healthy roots and clubroots were Proteobacteria at the phylum level, but the relative abundance differed. These results were in line with previous reports of many kinds of bacteria living in plant roots, including the phyla Proteobacteria, Actinobacteria and Bacteroidetes [19, 20]. In most studies, Proteobacteria are the predominant group of endophytic bacteria in various plant hosts [21, 22], suggesting they are suited to the ecological niche of plant tissue. Zhao also reported Proteobacteria as the dominant group of endophytic bacteria in the roots of oilseed rape (*Brassica napus*) [23]. Actinobacteria was the second dominant groups in healty roots and had high relative abundance, which is in line with Zhao’s results [23]. Some previous studies found that endophytic Actinomycetes had biocontrol capacity to inhibit some pathogens and also showed plant-growth-promotion traits [24–26]. In this study, Actinobacteria in healty roots maybe also have beneficial roles.

At the genus level, *Pseudomonas* dominated in clubroots, suggesting that this bacteria play an important role in the ecological niche. They may compete with *P. brassicae* for space and nutrition. Many previous studies verified that *Pseudomonas* possessed plant growth-promoting characteristics such as nitrogen fixation [27], production of plant hormones or antimicrobial substances, or inducing systemic plant defense responses [28]. The main genera in healthy roots was *Rhodanobacter*, which was also isolated from the roots of Spathiphyllum plants and had biocontrol activity against root rot fungal pathogen *Fusarium solani* [29, 30]. *Rhizobium* is widely distributed in plant root tissues and plays a role in nitrogen fixation for plant hosts [31–33]. In healthy roots and clubroots, we observed abundant *Rhizobium*, indicating that the bacteria probably fix nitrogen for tumorous stem mustard.

It was reported that endophytic bacterial community was altered by pathogen infection in many plants species such as grapevine [34], apple [12] and tomato [14]. Similarly in the present study, the differences in the endophytic bacterial community in healthy roots and clubroots were revealed by Hierarchical clustering analysis, PCA, NMDS and ANOSIM, suggesting that *P. brassicae* can restructure the endophytic bacterial community. To reveal how *P. brassicae* altered the community, we compared the physiological properties between healthy roots and clubroots and found marked differences in soluble sugar, soluble protein, methanol SOD and POD, showing that *P. brassicae* infection significantly changed the physiological characteristics.

*Plasmodiophora brassicae* is dependent on the nutrients, such as carbohydrates, from the host. Therefore, the pathogen upregulated the expression of sucrose synthase and starch synthase genes in clubroot [35], thus inducing accumulation of carbohydrates in clubroots, such as soluble sugars (hexoses and sucrose) and starch [36–39]. In our study, soluble sugar had the strongest correlation with the endophytic bacterial community in clubroot, suggesting that high concentration of soluble sugar could change the community. *Plasmodiophora brassicae* also absorbs amino acids and lipids from the galls of clubroot. Proteome studies demonstrate that the abundance of many proteins involved in plant physiological process alter in culbroots compared with healthy roots [40, 41]. The soluble protein increase in clubroot of Chinese cabbage infected by *P. brassicae* [42]. In our study, soluble protein increased in clubroots and correlated with the endophytic bacterial community, suggesting that soluble protein might also shape the community in clubroot. The rich nutritional substances in clubroot induced by *P. brassicae* infection promote some endophytic bacteria proliferation. For example, *Pseudomonas* possesses strong adaptation and ability of quick growth [43] and easily proliferate in the gall, which explained why *Pseudomonas* dominated in the endophytic bacterial coummunity in clubroots (Fig. 2b).

The methanol was also related to the endophytic bacterial community in clubroots. The previous studies showed that methanol production increased when plant cell wall endured mechanical wounding or other stresses such as pathogens or unsuitable temperature [44]. *Plasmodiophora brassicae* infection leads to root cell swellling and damages cell walls, which may promote root cells releasing more methanol. The content of methanol were markedly higher in clubroots than healthy roots, which probably impacted the endophytic bacterial community and promoted or inhibited some bacteria. For example, *Duganella* was the biomarker species in the community in clubroots and can utilize methanol as a carbon source [45]. Abundant *Duganella* in clubroots may be stimulated by methanol. The two-factor correlation network revealed that soluble sugar, soluble protein, methanol were related to endophytic bacteria, confirming soluble sugar, soluble protein, methanol restructured the endophytic bacterial community in clubroot.

SOD and POD are the antioxidative enzymes in plants that enhance plants tolerance to abiotic and biotic stress. In general, POD and SOD increase when plants are infected by pathogen [46, 47]. However, in the present study, SOD and POD in clubroots were lower than in healthy roots, suggesting that the normal physiological function might have been compromised by *P. brassicae* infection. Moreover, the db-RDA demonstrated that SOD and POD positively and negatively correlated with the endophytic bacterial community in healthy roots and clubroots, supporting the fact that *P. brassicae* infection inhibited the activity of SOD and POD.

## Conclusion

The discrimination in the endophytic bacterial community within the clubroots and healthy roots was revealed by high throughput sequencing. *Plasmodiophora brassicae* infection caused marked changes in physiological properties in clubroots. These physiological alterations inhibited or promoted some bacteria, and regulated the structure of the endophytic bacterial community. This study provides a new clue to understanding the interaction between pathogen and endophytic bacterial community in plants.

## Methods

### Samples

The clubroots of tumorous stem mustard were obtained at the harvest-stage (February 2, 2019) from three fields with distances 5 km in Fuling (29.21° N, 106.56° E) where clubroot disease had been found 20 years ago. The roots were classified as healthy roots (named R) and clubroots (named C). From one field, 30 plants were randomly selected and formed two groups (15 R samples and 15 C samples); thus, 6 groups containing 90 plants from 3 fields were named R1, C1, R2, C2, R,3 and C3. Soil particles attached to roots were removed by washing with tap water. The healthy roots with 0.5 cm diameter from healthy plants and clubroot galls with 1 cm diameter from diseased plants were cut off, surface sterilized by 70% (v/v) ethanol for 40 s, followed by 4% (w/v) sodium hypochlorite for 60 s and were finally rinsed three times in sterile distilled water. The surface-sterilized healthy roots and galls were cut with a sterilized razor and separated into two parts. One part was used for genomic DNA extraction and the part for physiological properties determination.

### Determination of physiologial properties of healthy roots and clubroots

The content of soluble sugar, soluble protein, POD, SOD, malondialdehyde and methanol in healthy roots and clubroots were detected according to the standard methods in Nanjing Cavenex Testing Technology Co. LTD. Soluble sugar, soluble protein and malondialdehyde were determined by the anthrone-sulfuric acid colorimetric method, the coomassie brilliant blue method and thiobarbituric acid method, respectively. SOD and POD were assessed by the NBT-illumination method and the guaiacol method, respectively. The methanol was measured by gas chromatography (GC-17A, Shimadzu, Kyoto, Japan).

### PCR amplification and 16S rRNA sequencing

Genomic DNA of healthy roots and clubroots was extracted using cetyltrimethylammonium bromide (CTAB). DNA concentration and purity were monitored on 1% w/v agarose gel. The bacterial V3 + V4 region of 16S ribosomal RNA gene was amplified by PCR for barcoded pyrosequencing using the primers (338F: 5′-ACTCCTACGGGAGGC AGCAG − 3′ and 806R: 5′-GGACTACHVGG GTWTCTAAT-3′) [48]. The forward primer 338F was linked to A-adaptor, a specific 8-bp multiplex identifier (MID) barcode, while the reverse primer 806R carried the B-adapter. The PCR conditions were: 95 °C for 2 min (one cycle), 95 °C for 30 s, 55 °C for 30 s, and 72 °C for 30 s (25 cycles), 72 °C for 5 min (one cycle). The sequencing was performed using an Illumina MiSeq sequencer (Majorbio Technology Co.,Ltd., China). The PCR reactions were performed in triplicate of 20 μL mixture containing 4 μL of 5 × FastPfu Buffer, 2 μL of 2.5 mM dNTPs, 0.8 μL of each primer (5 μM), 0.4 μL of FastPfu Polymerase and 10 ng of template DNA. The PCR products were confirmed by electrophoresis in agarose gel (2%) and resulted in amplified fragments of 500 bp that were further purified using an AxyPrep DNA Gel Extraction Kit (Axygen Biosciences, Union City, CA, USA) and quantified using QuantiFluor™-ST (Promega, USA) according to the manufacturer’s protocol. Purified amplicons were pooled and paired-end sequenced (2 × 300) on an Illumina MiSeq platform (Illumina, San Diego, USA) according to the standard protocols by Majorbio Bio-Pharm Technology Co. Ltd. (Shanghai, China).

### Bioinformatics processing and data analysis

The bioinformatics analysis was conducted on the free online Majorbio I–Sanger Cloud Platform (http://www.i-sanger.com/). Firstly, the raw sequences were processed using the Quantitative Insights Into Microbial Ecology (QIIME) package (v1.8) [49]. The low-quality sequences, such as primer and barcode sequence mismatches, sequences shorter than 50 bp, sequences containing ambiguous characters, PCR-based or sequencing errors and chimeras, were removed. The quality-filtered sequences were used to carry out identification of taxonomy of each OTU representative sequence by Unite (Release 7.2) software under the threshold of 97% identity [50]. Taxonomic assignment of representative sequences for each OTU was carried out on the basis of Silva (Release123 http://www.arb-silva.de) and the Ribosomal Database Project RDP (Release 11.3 http://rdp.cme.msu.edu/). The rarefaction curves, Shannon and Simpson index were used to indicate the community richness. Relative abundances of endophytic bacteria were assessed at the phylum, class, order, family, genus, species and OTU levels.

For β-diversity, the hierarchical cluster dendrograms (Bray-Curtis distance dissimilarities) were constructed according to OTU composition [51]. UniFrac-weighted PCA, NMDS and ANOSIM were performed to reveal the discrimination in the endophytic bacterial communities between healthy roots and clubroots using R 3.1.1 statistical software [52, 53]. LEfSe software was used to screen for the markedly different genera between healthy roots and clubroots for biomarker discovery [54]. Network analysis was performed to reveal the relationship among the top 50 OTUs within the endophytic bacterial communities by Networkx software based on Pearson’s rank correlation coefficients [55]. The db-RDA and two-factor correlation network were used to investigate relationships between the endophytic bacterial communities and physiological properties usimg Canoco statistical software (Version 5.0) with default parameter settings.

## Supplementary information

**Additional file 1: Table S1.** Taxonomy and distribution of the OTUs. Taxonomy at phylum, class, order, family, genus, species and OTU level. R, healthy roots. C, clubroots. The numbers in table cells are numbers of sequences of each OTU.

**Additional file 2: Figure S1.** Network analysis of the two endophytic bacterial communities in the healthy roots and clubroots. **a** Healthy roots. **b** Clubroots. Each node represents taxa affiliated at the OTU level, and the size of the nodes represents an average abundance of OTU. The lines represent the connections between each OTU. A red line indicates a positive correlation and a green line indicates a negative correlation.

## Data Availability

The raw reads of 16S MiSeq data were deposited in the NCBI Sequence Read Archive database (PRJNA631176).

## Abbreviations

PCA: UniFrac-weighted principal component analysis
NMDS: Non-metric multidimensional scaling
ANOSIM: Analysis of similarities
POD: Peroxidase
SOD: Superoxide dismutase
db-RDA: The distance-based redundancy analysis
OTUs: Operational taxonomic units
LEfSe: Discriminant Analysis Effect Size
MID: Multiplex identifier
CTAB: Cetyltrimethylammonium bromide
QIIME: Quantitative Insights Into Microbial Ecology
