# Supplementary material for: Physiological change alters endophytic bacterial community in clubroot of tumorous stem mustard infected by Plasmodiophora brassicae
Source: BMC Microbiol. 2020 Aug 6;20:244. doi: 10.1186/s12866-020-01930-4 (PMC7412676; doi:10.1186/s12866-020-01930-4)
Supplement: Supplementary file 2 — Additional file 2: Figure S1. Network analysis of the two endophytic bacterial communities in the healthy roots and clubroots. a Healthy roots. b Clubroots. Each node represents taxa affiliated at the OTU level, and the size of the nodes represents an average abundance of OTU. The lines represent the connections between each OTU. A red line indicates a positive correlation and a green line indicates a negative correlation. [file 12866_2020_1930_MOESM2_ESM.pdf]

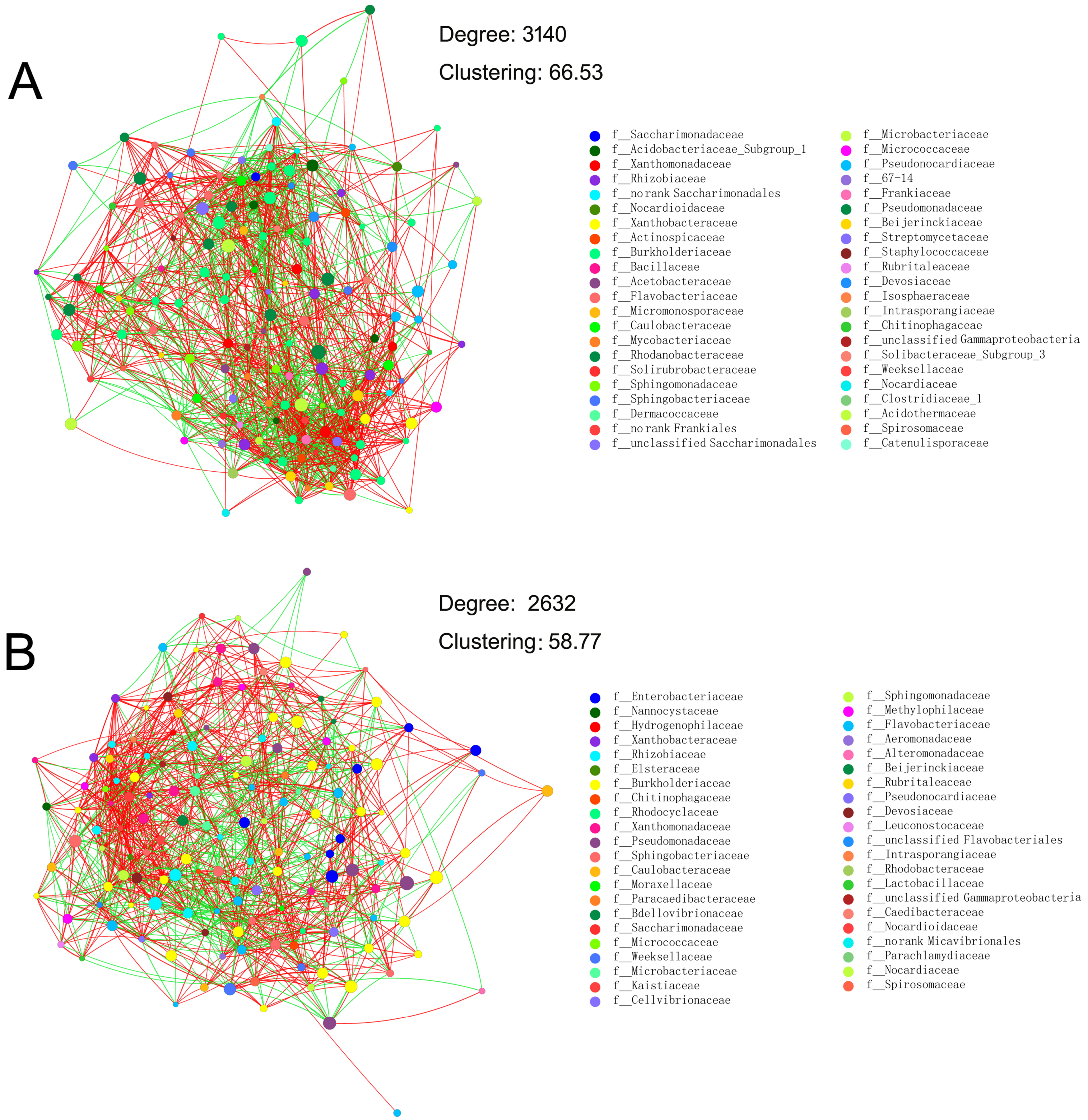
A
Degree: 3140
Clustering: 66.53
f__Saccharimonadaceae
f__Acidobacteriaceae_Subgroup_1
f__Xanthomonadaceae
f__Rhizobiaceae
f__norank Saccharimonadales
f__Nocardioidaceae
f__Xanthobacteraceae
f__Actinospicaceae
f__Burkholderiaceae
f__Bacillaceae
f__Acetobacteraceae
f__Flavobacteriaceae
f__Micromonosporaceae
f__Caulobacteraceae
f__Mycobacteriaceae
f__Rhodanobacteraceae
f__Solirubrobacteraceae
f__Sphingomonadaceae
f__Sphingobacteriaceae
f__Dermacoccaceae
f__norank Frankiales
f__unclassified Saccharimonadales
f__Microbacteriaceae
f__Micrococcaceae
f__Pseudonocardiaceae
f__67-14
f__Frankiaceae
f__Pseudomonadaceae
f__Beijerinckiaceae
f__Streptomycetaceae
f__Staphylococcaceae
f__Rubritaleaceae
f__Devosiaceae
f__Isosphaeraceae
f__Intrasporangiaceae
f__Chitinophagaceae
f__unclassified Gammaproteobacteria
f__Solibacteraceae_Subgroup_3
f__Weeksellaceae
f__Nocardiaceae
f__Clostridiaceae_1
f__Acidothermaceae
f__Spirosomaceae
f__Catenulisporaceae
B
Degree: 2632
Clustering: 58.77
f__Enterobacteriaceae
f__Nannocystaceae
f__Hydrogenophilaceae
f__Xanthobacteraceae
f__Rhizobiaceae
f__Elsteraceae
f__Burkholderiaceae
f__Chitinophagaceae
f__Rhodocyclaceae
f__Xanthomonadaceae
f__Pseudomonadaceae
f__Sphingobacteriaceae
f__Caulobacteraceae
f__Moraxellaceae
f__Paracaedibacteraceae
f__Bdellovibrionaceae
f__Saccharimonadaceae
f__Micrococcaceae
f__Weeksellaceae
f__Microbacteriaceae
f__Kaistiaceae
f__Cellvibrionaceae
f__Sphingomonadaceae
f__Methylophilaceae
f__Flavobacteriaceae
f__Aeromonadaceae
f__Alteromonadaceae
f__Beijerinckiaceae
f__Rubritaleaceae
f__Pseudonocardiaceae
f__Devosiaceae
f__Leuconostocaceae
f__unclassified Flavobacteriales
f__Intrasporangiaceae
f__Rhodobacteraceae
f__Lactobacillaceae
f__unclassified Gammaproteobacteria
f__Caedibacteraceae
f__Nocardioidaceae
f__norank Micavibrionales
f__Parachlamydiaceae
f__Nocardiaceae
f__Spirosomaceae
